# Exploring in vitro anti-proliferative and anti-inflammatory activities of Prasachandaeng remedy, and its bioactive compounds

**DOI:** 10.1186/s12906-022-03678-y

**Published:** 2022-08-11

**Authors:** Nuntika Prommee, Arunporn Itharat, Pakakrong Thongdeeying, Sunita Makchuchit, Weerachai Pipatrattanaseree, Adis Tasanarong, Buncha Ooraikul, Neal M. Davies

**Affiliations:** 1 Department of Applied Thai Traditional Medicine and Center of Excellence in Applied Thai Traditional Medicine Research (CEATMR), Faculty of Medicine, Thammasat University (Rangsit Campus), Klong Luang, Pathum Thani, 12120 Thailand; 2 Region Medicine Science Center 12 Songkhla, Department of Medical Science, Songkhla, 90110 Thailand; 3 Chulabhorn International College of Medicine, Thammasat University (Rangsit Campus), Klong Luang, Pathum Thani, 12120 Thailand; 4 Nephrology Unit, Faculty of Medicine, Thammasat University, Thammasat University (Rangsit Campus), Klong Luang, Pathum Thani, 12120 Thailand; 5 Department of Agricultural Food and Nutritional Science, Faculty of Agricultural Life and Environmental Sciences, University of Alberta, Edmonton, AB T6G2E1 Canada; 6 Bua Luang ASEAN, Thammasat University, Pathum Thani, 12120 Thailand; 7 Faculty of Pharmacy and Pharmaceutical Sciences, Katz Centre for Pharmacy and Health Research, University of Alberta, Edmonton, AB T6G2E1 Canada

**Keywords:** Thai traditional medicine, Cytotoxic activity, Anti-inflammatory, Tumor necrosis factor- α, Bioactive compounds

## Abstract

**Background:**

Prasachandaeng (PSD) remedy has been empirically used in Thai traditional medicine to treat fever in bile duct and liver and cancer patients through Thai folk doctors. However, there have been no scientific reports on the bioactive compounds and bioactivities related to inflammation-associated carcinogenesis or cytotoxicity against cancer cell lines. In this study, we investigated the chemical content of the remedy, and evaluated its cytotoxic activity against two cancer cell lines in comparison with a non-cancerous cell line and determined tumor necrosis factor-alpha (TNF-α) production in a murine macrophage cell line (RAW 264.7) to evaluate anti-inflammatory activity. A novel HPLC method was used for quality control of its chemical content.

**Methods:**

Pure compounds from the EtOH extract of *D. cochinchinensis* were isolated using bioassay-guided fractionation and chemical content of the PSD remedy was determined using HPLC. The cytotoxic activity against the hepatocarcinoma cell line (HepG2) and cholangiocarcinoma cell line (KKU-M156), in comparison with non-cancerous cell line (HaCaT), were investigated using antiproliferative assay (SRB). The anti-inflammatory activity measured by TNF-α production in RAW 264.7 was determined using ELISA.

**Results:**

All crude extracts and isolated compounds exhibited significant differences from vincristine sulfate (^****^p < 0.0001) in their cytotoxic activity against HepG2, KKU-M156, and HaCaT. The PSD remedy exhibited cytotoxic activity against HepG2 and KKU-M156 with IC_50_ values of 10.45 ± 1.98 (SI = 5.3) and 4.53 ± 0.74 (SI = 12.2) µg/mL, respectively. Some constituents from *C. sappan*, *D. cochinchinensis*, *M. siamensis*, and *M. fragrans* also exhibited cytotoxic activity against HepG2 and KKU-M156, with IC_50_ values less than 10 µg/mL. The isolated compounds, i.e., Loureirin B (1), 4-Hydroxy-2,4’-dimethoxydihydrochalcone (2), and Eucomol (3) exhibited moderate cytotoxicity against two cancer cell lines. None of the crude extracts and isolated compounds showed cytotoxicity against HaCaT. *D. cochinchinensis* and PSD remedy exhibited higher anti-inflammatory activity measured as TNF-α production than acetaminophen.

**Conclusion:**

The findings provide evidence of bioactivity for EtOH extracts of PSD remedy and the isolated compounds of *D. Cochinchinensis.* The results consistent the use clinical activity and use of PSD remedy as a antipyretic treatment for liver and bile duct cancer patients by Thai traditional practitioners.

## Background

In Thailand's contemporary society, the incidence of primary hepatocellular carcinoma is very high. The occurrence of cancer of the liver and bile duct ranks the highest among male patients (19.5%) and seventh among females (3.8%) [1]. Furthermore, hepatocellular carcinoma is the third leading cause of death world-wide [2]. The percentage of hepatocellular carcinoma and cholangiocarcinoma varies greatly between different regions of Thailand. The frequency rate of new cases of cholangiocarcinoma in the Northeast region has increased to the highest in the world [3]. The major risk factors are chronic infections of the hepatitis B virus (HBV), hepatitis C virus (HCV), and high exposure to aflatoxins [4]. Traditionally, cancer patients in Thailand have used complementary medicine for the treatment of chronic diseases, such as degenerative disease, inflammatory, and pain disorders as well as cancers [5].

The current study involves an anti-cancer proliferative activity that has been used for screening the cytotoxicity of herbal medicine extracts and bioactive compounds. The SRB assay is related to the mechanism of antiproliferation of cancer cells [6]. Tumor necrosis factor-alpha (TNF-α) is a pro-inflammatory cytokine that can be secreted by inflammatory cells, which involves inflammation-associated carcinogenesis. The cytotoxic activity and anti-inflammatory activity through inhibition of TNF-α production is a means of evaluating cancer prevention and therapy [7].

The National List of Essential Medicine (NLEM) of Thailand lists many herbal combinations for treating several diseases, such as headache, dyspepsia, gastritis, fever, etc. Prasachandaeng (PSD) remedy, is an antipyretic medicine used to treat toxic or chronic fever in both adults and children [8]. PSD remedy has been used by Thai folk doctors to treat toxic (chronic) fever in liver and bile duct cancer patients. Thai traditional wisdom describes toxic or chronic fever as the origin of cancer. This ethnotraditional description agrees with modern Western medicine where inflammation or continual fever can lead to cancer [2]. A Thai traditional textbook, Ka-Sai scripture, has a chapter on chronic and cancer symptoms that explains degenerative diseases which lead to chronic disease such as cancer [9]. The causes of chronic diseases, according to Thai traditional medicine (TTM), include food poisoning, behavioral disorders, chronic inflammation, and infection. Diseases related to hepatocellular carcinoma and cholangiocarcinoma recorded in the Thai traditional scripture, Ka-Sai, and scientific evidence of utility of Thai traditional remedies are consistent with several symptoms described in Western medicine, such as weight loss, anemia, pale skin, abdominal pain, lack of appetite, insomnia, yellow urine, chronic fever, constipation, and unhealthy body and mind (Table 1). However, the PSD remedy has not been scientifically investigated with respect to bioactive compounds and bioactivities related to inflammation-associated carcinogenesis. Therefore, the aims of this study were to isolate compounds using a bioassay-guided fractionation from the main ingredients of PSD remedy using HPLC analysis for quality control. and to investigate cytotoxicity activity against two cancer cell lines, i.e., hepatocarcinoma (HepG2) and cholangiocarcinoma (KKU-M156) in comparison with a non-cancerous cell line (HaCaT) using the antiproliferative assay (SRB assay). The anti-inflammatory activity measured as inhibition of TNF-α production in the murine macrophage cell line (RAW 264.7) was also investigated using an enzyme-linked immunosorbent assay (ELISA).

**Table 1:** The comparison of symptoms of liver and bile duct cancer in Thai traditional medicine (TTM) and modern medicine

Symptoms	Thai traditional medicine^a^	Modern medicine^b^
Anemia	**✓**	**-**
Pale skin	**✓**	**-**
Insomnia	**✓**	**-**
Constipation	**✓**	**-**
Chronic fatigue	**✓**	**-**
Jaundice	**✓**	**✓**
Itching	**-**	**✓**
Light-colored	**✓**	**✓**
Greasy stools	**-**	**✓**
Dark urine	**-**	**✓**
Abdominal pain	**✓**	**✓**
Loss of appetite	**✓**	**✓**
Weight loss	**✓**	**✓**
Nausea and vomiting	**✓**	**✓**
Chronic fever	**✓**	**✓**

(**✓**) indicates found as a symptom, (-) indicates not found as a symptom

^a^ Thai traditional medical textbook, The Rehabilitation Foundation for Thai Traditional Medicine and Ayuraved Thamrong School, 2007

^b^ American cancer society, 2020

## Methods

### Chemicals and reagents

Ethanol (EtOH), 95%, was purchased from C.M.J. Anchor company, Thailand. Acetic acid, Sulfuric acid and Trichloroacetic acid (TCA) were purchased from Merck, Germany. Analytical grade reagents, i.e., hexane, chloroform (CHCl_3_), ethyl acetate (EtOAc), methanol (MeOH), dimethylsulfoxide (DMSO), hydrochloric acid (HCl) were purchased from RCI Labscan, Thailand. Dulbecco’s modified eagle medium (DMEM), fetal bovine serum (FBS), minimum essential medium (MEM), penicillin–streptomycin (P/S), and phosphate-buffered saline (PBS) were purchased from Biochrom, Germany. Sodium bicarbonate (NaHCO_3_) was purchased from BHD, United Kingdom. Sodium hydroxide (NaOH) was purchased from Univar, Australia. Sulforhodamine B sodium salt, Tris [hydroxymethyl] aminoethane, HEPES buffer solution, nutrient mixture F-12 Ham (HAM’s F12), and lipopolysaccharide (LPS) were purchased from Sigma-Aldrich, USA. Trypan blue stain 0.4% and trypsin–EDTA were purchased from Gibco, USA. Silica Gel 60 (particle size 0.063–0.200 mm) for vacuum liquid chromatography (VLC), Silica Gel 60 (particle size 0.040–0.063 mm) for column chromatography (CC), and thin layer chromatography (TLC) silica gel 60 F254 were purchased from Merck, Germany. Anisaldehyde reagent was purchased from Fluka, Switzerland. NMR spectra were obtained with a Bruker Avance 400 spectrometer at 400 and 500 MHz for ^1^H NMR and 100 and 125 MHz for ^13^C NMR. The chemical shifts were recorded in *δ*_H,_ *δ*c (ppm) in CDCl_3_. Rotary evaporator was purchased from Buchi, Switzerland. UV spectrophotometer was purchased from SHIMADZU, Japan. CO_2_ incubator was purchased from Shellab, USA. Laminar flow cabinet was purchased from Boss tech, Thailand. Microplate reader was purchased from Bio Tek instrument, USA.

### Identification of plant ingredients of PSD remedy

Plant ingredients of PSD remedy were harvested from several regions of Thailand. Species identification was approved by the Herbarium of Southern Center of Thai Medicinal Plants at the Faculty of Pharmaceutical Science, Prince of Songkhla University, Songkhla, Thailand as shown in Table 2. All plant materials were carried out according to the standard of quality control of plant materials published earlier [10].

**Table 2:** The general information of the plant ingredients from Prasachandaeng (PSD) remedy

Plant species (Family)	Voucher specimen number	Part used	Collected from	Proportion (%w/w)
*Bouea macrophylla* Griff. (Anacardiaceae)	SKP 009 02 02 01	Twig	Nakhonnayok	6.25
*Caesalpinia sappan* L. (Leguminosae)	SKP 098 03 19 01	Heartwood	Sukhothai	6.25
*Citrus aurantiifolia* (Christm.) Swingle (Rutaceae)	SKP 166 03 01 01	Twig	Nakhonpathom	6.25
*Dracaena cochinchinensis* (Lour.) S.C. Chen (Dracaenaceae)	SKP 065 04 12 01	Heartwood	Phitsanulok	50.00
*Heliciopsis terminalis* (Kurz) Sleumer (Proteaceae)	SKP 187 19 18 01	Twig	Chiangmai	6.25
*Jasminum sambac* (L.) Aiton (Oleaceae)	SKP 129 10 19 01	Flower	Nakhonpathom	1.56
*Kaempferia galanga* L. (Zingiberaceae)	SKP 206 11 07 01	Rhizome	Nakhonpathom	6.25
*Ligusticum chuanxiong* Hort. (Umbelliferae)	SKP 199 12 19 01	Rhizome	China	6.25
*Mammea siamensis* T.Anderson. (Calophyllaceae)	SKP 083 13 19 01	Flower	Chantaburi	1.56
*Mesua ferrea* L. (Calophyllaceae)	SKP 083 13 06 01	Flower	Phichit	1.56
*Myristica fragrans* Houtt. (Myristicaceae)	SKP 121 13 06 01	Heartwood	Indonesia	6.25
*Nelumbo nucifera* Gaertn. (Nelumbonaceae)	SKP 125 14 14 01	Pollen	Nakhonpathom	1.56

### Preparation and extraction

Plant ingredients were cleaned, sliced to small pieces, and dried at 45 °C in a hot air oven. The plant ingredients were weighed and mixed according to the PSD remedy proportion as shown in Table 2. The PSD remedy powder (1,000 g) was macerated with EtOH (5,000 mL) for 72 h and filtered through a Whatman filter paper No. 1 and re-macerated twice. The combined extract was dried using a rotary evaporator. Each crude powder of plant (200 g) was extracted with the same method as above. All crude extracts were kept at -20 °C before bioactivities testing and chemical analysis.

### Isolation of compounds from *Dracaena cochinchinensis* (Lour.) S.C. Chen

In this study, *Dracaena cochinchinensis* (Lour.) S.C. Chen was the main herbal ingredient of PSD remedy constituting 50% w/w of all proportions and the EtOH extract of *D. cochinchinnesis* (DC95) showed the most potent cytotoxic activity against two cancer cell lines (HepG2 and KKU-M156). Therefore, the DC95 was chosen for the isolation of pure compounds by bioassay-guided fractionation. DC95 (70 g) was chromatographed in a vacuum liquid chromatography (VLC) using silica gel 60 (300 g) with gradient elution which provided five fractions as follows: hexane (Fraction 1: 2,000 mL), hexane: CHCl_3_ (Fraction 2: 1:1, 2,000 mL), chloroform (Fraction 3: 2,000 mL), CHCl_3_: MeOH (Fraction 4: 1:1, 2,000 mL) and MeOH (Fraction 5: 2,000 mL), respectively. The percentage of yield as %w/w of the starting weight of crude extracts are shown in Table 3. Based on the cytotoxic activity against HepG2 and KKU-M156 cell lines by SRB assay, F3 was chosen for the bioassay-guided isolation as it demonstrated highest cytotoxicity against two types of cancer cell lines (HepG2 and KKU-M156) with the IC_50_ values of 43.14 ± 1.50 and 42.26 ± 1.07 µg/mL, respectively. F1-5 fractions showed no cytotoxicity against a human normal cell line (HaCaT) with the IC_50_ > 50 µg/mL. Fraction 3 (7.0 g) was chromatographed in a column chromatography (CC) on silica gel 60 (150 g) with gradient elution to give six fractions as followed: hexane:EtOAC (6:4, 5,000 mL), hexane:EtOAC (7:3, 500 mL), hexane:EtOAC (1:1, 500 mL), EtOAC (500 mL), EtOAC:MeOH (1:1, 500 mL), and MeOH (500 mL), respectively. The eluent was collected and examined by thin layer chromatography (TLC) with UV 254 detector at 356 nm, and was sprayed with anisaldehyde reagent. The structure of the isolated compounds were identified by ^1^H-NMR, ^13^C-NMR, DEPT135, DEPT90, COSY, NOESY, HSQC, and HMBC. Subfraction Fr.11 (416 mg) was also purified by CC on silica gel 60 (50 g) and eluted with hexane: CHCl_3_ (1:9) to afford a 2 (8.4 mg, 0.12% w/w of crude extract) as a white amorphous powder and a 3 (10.5 mg, 0.15% w/w of crude extract) as colorless crystal. The subfraction Fr.15 (146 mg) was also purified by recrystallization with hexane: CHCl_3_ (7:3) to provide a 1 (6.8 mg, 0.10%w/w of crude extract) as a white amorphous powder.

**Table 3:** The IC_50_ µg/mL ± SEM of cytotoxicity against two cancer cell lines and one non-cancerous cell line of VLC isolated fractions, isolated pure compounds and ethanolic extract of *D.cochinchinensis*) and comparison with a standard chemotherapeutic drug (Vincristine sulfate) (*n* = 3)

Sample	Code	%yield (w/w)	IC_50_ µg/mL ± SEM and Selective index (SI)		
			**HepG2**	**KKU-M156**	**HaCat**
Fraction 1 (hexane)	F1	0.19	NT	NT	NT
Fraction 2 (hexane: CHCl_3_, 1:1)	F2	2.31	> 100^****^	> 100^****^	> 100^****^
Fraction 3 (CHCl_3_)	F3	14.57	43.14 ± 1.50^****^ (SI = 2.2)	42.26 ± 1.07^****^ (SI = 2.2)	92.96 ± 2.22^****^
Fraction 4 (CHCl_3_: MeOH, 1:1)	F4	78.65	51.95 ± 0.37^****^ (SI = 1.8)	61.51 ± 5.08^****^ (SI = 1.5)	95.01 ± 0.42^****^
Fraction 5 (MeOH)	F5	4.27	> 100^****^	> 100^****^	> 100^****^
Loureirin B **(1)**	P1	0.10	20.02 ± 0.46^****^ (SI = 4.3)	21.26 ± 3.17^***^ (SI = 4.1)	86.12 ± 2.13^****^
4-Hydroxy-2,4’-dimethoxydihydrochalcone **(2)**	P2	0.12	20.71 ± 0.49^****^ (SI = 3.2)	33.21 ± 2.10^****^ (SI = 2.0)	66.00 ± 0.68^****^
Eucomol **(3)**	P3	0.15	25.76 ± 1.56^****^ (SI = 1.8)	7.12 ± 0.56^****^ (SI = 6.5)	46.55 ± 0.88^****^
EtOH of *D.cochinchinensis*	DC95	10.45	7.72 ± 1.87^****^ (SI = 5.2)	5.27 ± 5.01^****^ (SI = 7.7)	40.47 ± 0.39^****^
Vincristine sulfate	-	-	0.012 ± 0.0005 (SI = 0.00058)	0.0026 ± 0.001 (SI = 0.0026)	0.000007 ± 0.00

NT mean not tested due to weight of extract less than 5 mg. *SI* Selective index calculated by IC_50_ of non-cancerous cells / IC_50_ of cancer cells. Data were presented as mean ± SEM and analyzed by one-way ANOVA and Dunnett’s multiple comparison tests. Significant different presented the ****p < 0.0001 compared with a standard drug (Vincristine sulfate) in corresponding cell line

### Determination of the isolated compounds in Prasachandaeng remedy

#### Instruments

The HPLC method followed a protocol previously described [11, 12] with only a slight modification. It was performed on an Agilent® 1200 HPLC system (Agilent Technologies, USA) composing of a solvent degasser (G1322A), a quaternary solvent pump (G1311A), an autosampler (G1329A), a column oven (G1316A), and a photodiode array detector (G1315D). The chromatographic data were processed by the Chemstation® software revision B.04.01 SP1. The reversed-phase C18 column was Phenomenex® Luna SU C18(2)/100(A), column size 150 × 4.6 mm.

### High performance liquid chromatography analysis

The HPLC method was modified from Pipatrattanaseree et al., 2019 [12]. The EtOH extract of PSD remedy was prepared at a concentration of 10 mg/mL. An accurately weighed extract was dissolved with methanol and sonicated for 15 min. The solution of each isolated compound was prepared at a concentration of 1 mg/mL in methanol for the identification of chromatograms and quantitative analysis of chemical contents. All samples were filtered through 0.45 micron before analysis with the HPLC system. The serial dilution of three marker compounds of PSD remedy, i.e., Loureirin B (1), 4-Hydroxy-2,4’-dimethoxydihydrochalcone (2), and Eucomol (3) were injected into HPLC, and the calibration curves constructed according to their responses. All standard curves demonstrated linearity with the r^2^ > 0.99 within the linear range. All quantitatively determined data from the isolated compounds in PSD remedy was expressed as the mean ± standard error of the means (SEM) of at least three independent experiments.

### Chromatographic system

The solvent system consisted of a gradient mobile phase of water (A) and acetonitrile (B) which was programmed as follows: 0–5 min, (A:B; 95%:5%), 5–55 min, (A:B; 30%:70%), and 55–60 min, (A:B; 90%:10%), respectively. The flow rate was set at 1 mL/min and the pressure limit was 400 bars. The samples were injected into the HPLC system and detected with diode array detector at 280 nm wavelength.

### Cytotoxic activity using antiproliferative assay (Sulforhodamine B assay)

#### Cell culture

Hepatocellular carcinoma (HepG2; ATTC No. HB-8065) was cultured in Minimum Essential Media (MEM) supplemented with 10% heated-inactivated fetal bovine serum (FBS) and 1% penicillin–streptomycin (P/S). Cholangiocarcinoma cell line (KKU-M156) was cultured in HAM's F12 supplemented with 10% FBS, 1% P/S, and 12.5 mM HEPES. In addition, one non-cancerous human keratinocyte cell line (HaCaT; No. 300493-SF) was cultured in Dulbecco's Modified Eagle Medium (DMEM) supplemented with 10% FBS and 1% P/S [13].

### In vitro Sulforhodamine B assay

**Fig. 1:**
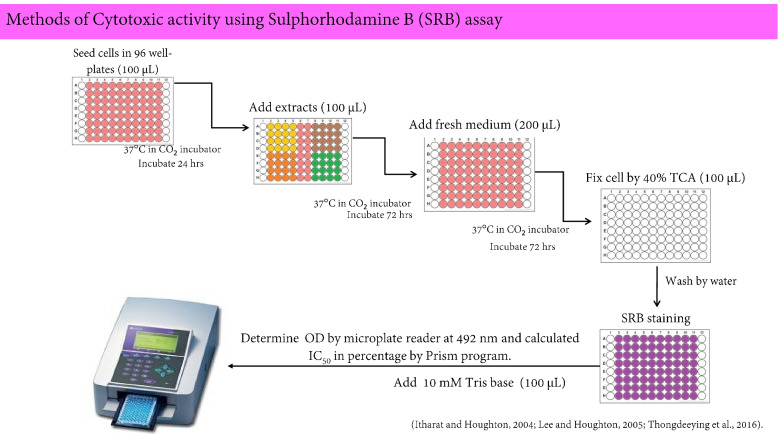
The In vitro Sulforhodamine B assay protocol

This assay followed the previously described protocol [5, 13]. The various concentrations (1, 10, 50, and 100 µg/mL) of the crude extracts were investigated against two human cancer cell lines and one non-cancerous cell line. The cell lines were washed with PBS and the cells were detached with 0.025% trypsin–EDTA to make a single cell suspension. A 5 mL medium was then added to the flask to stop the trypsin–EDTA activity. The viable cells were counted by trypan blue exclusion in a haemocytometer. A single-cell suspensions density of HepG2, KKU-M156, and HaCaT were diluted with each medium to give optimal densities of 2 × 10^3^, 3 × 10^3^, and 8 × 10^3^ cells/well, respectively. The 100 µL/well of these cell suspensions were seeded in 96-well plates and incubated at 37 °C with 5% CO_2_ at 95% humidity for 24 h. Then, 100 µL of a sample solution was added to each well. The control was the medium mixed with 2% DMSO. The 96-well plates were incubated at 37 °C with 5% CO_2_ at 95% humidity for 72 h. The mixture in the well was removed and washed with 200 µL fresh medium. The 96-well plate was further incubated for 72 h, then the cell in 96-well plates was fixed with 40%TCA and washed with water five times. Finally, the fixed cell in the 96-well plate was strained with Sulforhodamine B sodium salt and the percentage of inhibition of cell growth was measured colorimetrically using the SRB assay [5, 13, 14]. The herbal extract and pure compounds were considered to have potent cytotoxicity if the IC_50_ values were ≤ 20 and ≤ 4 µg/mL, respectively [15]. The % inhibition of cell growth was calculated by the equation shown below, and the IC_50_ values were calculated using the Prism program. The protocol of in vitro Sulforhodamine B assay is shown in Fig. 1.

$$\%\;\mathrm I\mathrm n\mathrm h\mathrm i\mathrm b\mathrm i\mathrm t\mathrm i\mathrm o\mathrm n\;\mathrm o\mathrm f\;\mathrm c\mathrm e\mathrm l\mathrm l\;\mathrm g\mathrm r\mathrm o\mathrm w\mathrm t\mathrm h=\left[\left({\mathrm M\mathrm e\mathrm a\mathrm n\;\mathrm o\mathrm f\;\mathrm O\mathrm D}_{\mathrm{control}}-{\mathrm M\mathrm e\mathrm a\mathrm n\;\mathrm o\mathrm f\;\mathrm O\mathrm D}_{\mathrm{sample}}\right)\div{\mathrm M\mathrm e\mathrm a\mathrm n\;\mathrm o\mathrm f\;\mathrm O\mathrm D}_{\mathrm{sample}}\right]\times100$$

where: OD _control_ = OD of medium with 2% of DMSO and OD _sample_ = OD of crude extract.

The Selectivity index (SI) exhibited the ratio of the half-maximal inhibitory concentration (IC_50_) of non-cancerous cell line and the half-maximal inhibitory concentration (IC_50_) of cancer cell line [16]. Additionally, when the SI value was determined to be higher than three it was chosen as a prospective in vitro anti-proliferative sample [17].

$$\mathrm S\mathrm e\mathrm l\mathrm e\mathrm c\mathrm t\mathrm i\mathrm v\mathrm i\mathrm t\mathrm y\;\mathrm i\mathrm n\mathrm d\mathrm e\mathrm x\left(\mathrm{SI}\right)=\mathrm{IC}50\;\mathrm o\mathrm f\;\mathrm n\mathrm o\mathrm n-\mathrm c\mathrm a\mathrm n\mathrm c\mathrm e\mathrm r\mathrm o\mathrm u\mathrm s\;\mathrm c\mathrm e\mathrm l\mathrm l\;\mathrm l\mathrm i\mathrm n\mathrm e\div\mathrm{IC}50\;\;\mathrm o\mathrm f\;\mathrm c\mathrm a\mathrm n\mathrm c\mathrm e\mathrm r\;\mathrm c\mathrm e\mathrm l\mathrm l\;\mathrm l\mathrm i\mathrm n\mathrm e$$

### Anti-inflammatory activity on inhibition of TNF- α production

**Fig. 2:**
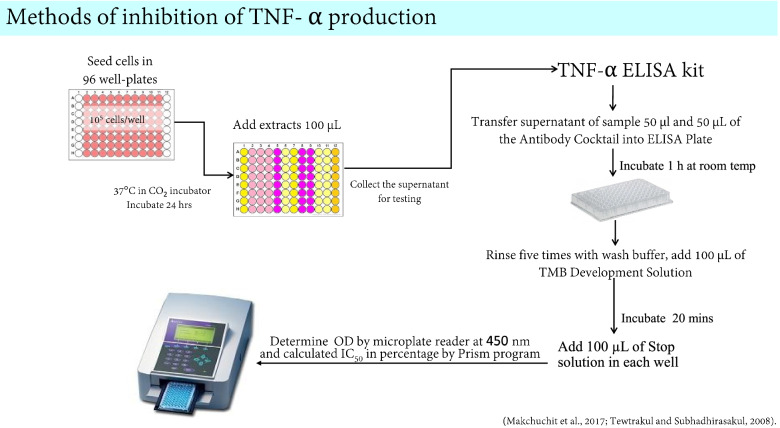
The anti-inflammatory activity through inhibition of TNF- α production protocol

The tumor necrosis factor-alpha (TNF-α) is the principal mediator of inflammation in response to gram-negative bacteria. It is mainly produced by LPS-activated mononuclear phagocytes. The TNF- α ELISA can quantify TNF-α in the supernatant of cell culture medium [18]. The assay was carried out using the TNF-alpha ELISA kit (Thermo Fisher® scientific, USA). The murine macrophage cell line (RAW 264.7) was cultured in DMEM medium containing 10% heat-inactivated FBS, 10^4^ µg/mL P/S. Firstly, the viable cells were counted using trypan blue exclusion in a haemocytometer. The single cell suspension of murine macrophage cell line was diluted with the medium to provide an optimal density of 10^5^. The cell suspension was seeded, in a 100 µL/well, in 96-well plates and incubated at 37 °C with 5% CO_2_ atmosphere at 95% humidity for 24 h. Secondly, 100 µL of fresh medium containing 5 ng/mL of lipopolysaccharide and 100 µL at 100 µg/mL of test sample for screening. Besides, if the test sample shows the %inhibition of TNF- α production of more than 50%, we will investigate the various concentrations at 1, 10, 50, and 100 µg/mL of test sample for calculation of IC_50_. Then, the test sample was added and incubated for 24 h. The control included 2% DMSO solution mixed with the medium, in place of the test samples in the analyses. This protocol was followed according to TNF-α ELISA kit (Thermo Fisher® scientific, USA). The various reagents, such as biotinylated detection antibody, streptavidin-HRP, HRP diluent, wash buffer, chromogen stop solution, were prepared before starting the experiment. Firstly, 50 µL of all samples or standard were added to appropriate wells. Secondly, 50 µL of the antibody cocktail was added to each well and the plate sealed and incubated for 1 h at room temperature on a plate shaker. Then, the wells were washed with wash buffer. Lastly, 100 µL of TMB development solution was added to each well and incubated for 10 min in the dark on a plate shaker set to 400 rpm and 100 µL of stop solution was added to each well. The 96-well plate was shaken for 1 min and then incubated for 20 min. The concentrations of TNF- α in the wells were measured with a microplate reader at 450 nm [19, 20]. The % inhibition of TNF-α production was calculated using the equation below, and the IC_50_ values were calculated using the Prism program. The protocol of the anti-TNF- α production is shown in Fig. 2.

$$\mathrm{\%Inhibition}= \left[\left({\mathrm{Mean of OD}}_{\mathrm{control}}-{\mathrm{Mean of OD}}_{\mathrm{sample}}\right)/{\mathrm{Mean of OD}}_{\mathrm{control}}) \right]100$$

Where: Mean of OD_control_ = Mean of OD_control (-LPS)_ – Mean of OD_control (+LPS)_, Mean of OD_sample_ = Mean of OD_sample (+LPS)_ – Mean of OD_control (+LPS)_.

### Statistical analysis

All data are expressed as the mean ± standard error of the means (SEM) of at least three independent experiments. The % inhibition values were calculated using the Microsoft Excel program. The IC_50_ values and statistical significance were calculated using the GraphPad Prism software, version 8.0.1 (San Diego, CA, USA). Statistical differences were analyzed by one-way ANOVA, followed by Dunnett’s multiple comparison tests using. The statistical significance was assessed at *p < 0.05, ^**^ *p* < 0.01, ^***^ *p* < 0.001, and ^****^ *p* < 0.0001.

## Results

### Structure elucidation of isolated pure compound and their bioactivity

The results of the percentage of yield (%w/w) and cytotoxic activity are shown in Table 3. Fraction 3 (F3) was chosen for the bioassay-guided fractionation as it exhibited potent cytotoxicity against two cancer cell lines (HepG2 and KKU-M156) in comparison with human non-cancerous cell line (HaCaT) with the IC_50_ value of 43.14 ± 1.50 (SI = 2.2) and 42.26 ± 1.07 (SI = 2.2) µg/mL, respectively. F2, F4, and F5 did not show cytotoxic activity against two cancer cell lines and one type of non-cancerous cell line. Three isolated compounds from the EtOH extract of *D. cochinchinensis*, were Loureirin B (1), 4-Hydroxy-2,4’-dimethoxydihydrochalcone (2), and Eucomol (3). The ^1^H and ^13^C NMR spectral data of compound 2 were closely related to compound 1 (Table 4), except the methoxy group at C-6 was absent.

**Table 4:** NMR spectral data of compounds 1 and 2 in CDCl_3_

**Position**	**1**			**2**		
	***δ***_**C**_	***δ***_**H**_** (*J* in Hz)**	HMBC (*δ*_H_ → *δ*_C_)	***δ***_**C**_	***δ***_**H**_** (*J* in Hz)**	HMBC (*δ*_H_ → *δ*_C_)
1	109.7			122.0		
2	158.7			159.5		
3	90.4	6.12 (s)	C-1, C-4, C-5, C-β	98.7	6.44 (d, 2.3)	C-1, C-2, C-5
4	159.5			158.4		
5	90.4	6.12 (s)	C-1, C-3, C-4, C-β	104.1	6.41 (dd, 8.2, 2.3)	C-1, C-3
6	158.7			130.3	7.07 (d, 8.2)	C-2, C-3, C-4, C-β
1′	129.9			130.2		
2′	130.8	7.92 (d, 8.7)	C-1′, C-3′, C-4′, C-6′, C = O	130.8	7.91 (d, 8.8)	C-1′, C-3′, C-4′, C-6′, C = O
3′	115.2	6.87 (d, 8.7)	C-1′, C-2′, C-5′	115.3	6.88 (d, 8.8)	C-1′, C-4′, C-5′
4′	160.2			160.2		
5′	115.2	6.87 (d, 8.7)	C-1′, C-3′, C-6′	115.3	6.88 (d, 8.8)	C-1′, C-3′, C-4′
6′	130.8	7.92 (d, 8.7)	C-1′, C-2′, C-4′, C-5′, C = O	130.8	7.91 (d, 8.8)	C-1′, C-2′, C-4′, C = O
α	38.4	3.04 (m)	C-1, C-β, C = O	38.9	3.17 (m)	C-1, C-β, C = O
β	18.6	2.97 (m)	C-1, C-6, C-α, C = O	25.4	2.95 (m)	C-1, C-2, C-6, C-α C = O
2-OCH_3_	55.6	3.80 (s)	C-2	55.4	3.78 (s)	C-2
4-OCH_3_	55.3	3.76 (s)	C-4	55.3	3.78 (s)	C-4
6-OCH_3_	55.6	3.80 (s)	C-6			
C = O	200.3	-	-	199.3		

Note: Compound 1; 1H and 13C data were recorded at 500 and 125 MHz and Compound 2; 1H and 13C data were recorded at 400 and 100 MHz

**Fig. 3:**
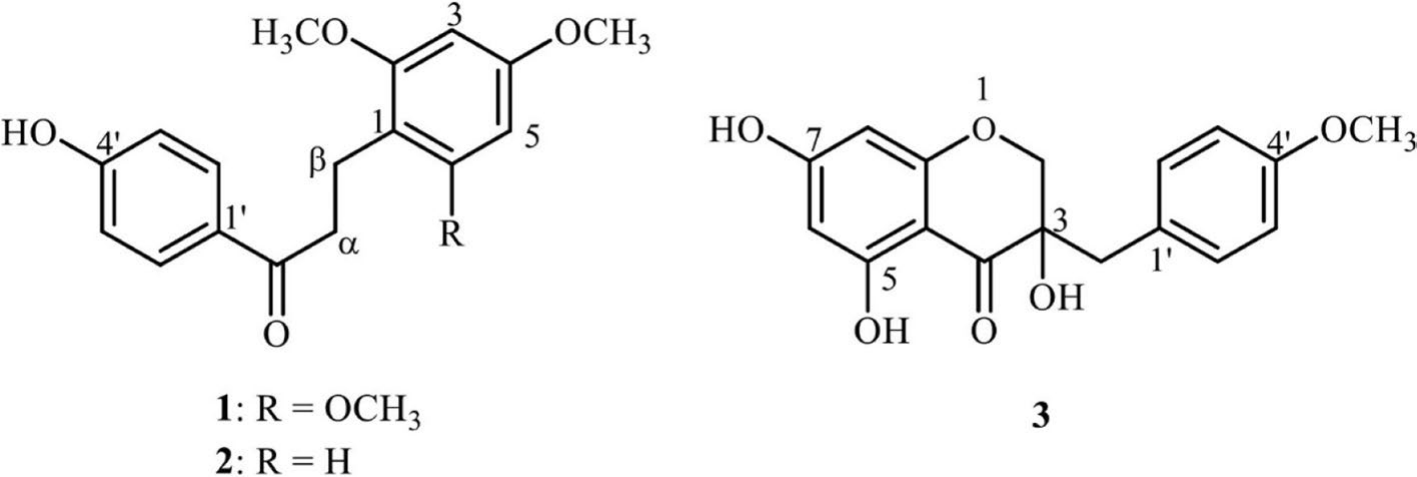
The chemical structure of three isolated compounds from the heartwood of *Dracaena cochinchinensis* (Lour.) S.C. Chen

Loureirin B (1) was obtained as a white amorphous powder. The ^1^H and ^13^C data were recorded at 500 and 125 MHz in CDCl_3_, respectively. The NMR spectrum of compound 1 displayed the presence of *para*-disubstituted aromatic protons at *δ* 7.92 and 6.87 (each d, *J* = 8.7 Hz, H-2´, H-6´ and H-3´, H-5´, respectively) and doublet *meta* proton at *δ* 6.12 (d, *J* = 2.3 Hz, H-3, and H-5). Three methyl protons at *δ* 3.80 (6H, s) and 3.76 (3H, s) suggest the presence of methoxy groups at 2-OCH_3_, 6-OCH_3_, and 4-OCH_3_, respectively. The appearance of the carbonyl group at *δ* 200.3 and two aliphatic protons at *δ* 3.04 (2H, H-α) and 2.97 (2H, H-β) displayed a similar signal pattern to those of retrodihydrochalcone [21]. In the HMBC spectra (Table 4 and Fig. 3), the position of H-α (*δ* 3.04) shows correlations with C-1 (*δ* 109.7), C-β (*δ* 18.6) and C = O (*δ* 200.3). The aromatic proton H-2´ (*δ* 7.92) correlated with C-1´ (*δ* 129.9), C-3´ (*δ* 115.2), C-4´ (*δ* 160.2), C-6´ (*δ* 130.8) and C = O (*δ* 200.3), and the aromatic proton H-3 (*δ* 6.12) correlated with C-1 (*δ* 109.7), C-4 (*δ* 159.5), C-5 (*δ* 90.4) and C-β (*δ* 18.6). These spectral data identified compound 1 as Loureirin B (1-(4-hydroxyphenyl)-3-(2,4,6-trimethoxyphenyl)propan-1-one), which structure has been described in a previous report [21]. Compound 1 exhibited potent cytotoxicity against HepG2 and KKU-M146 with the IC_50_ values of 20.02 ± 0.46 and 21.26 ± 3.17 µg/mL, respectively (Table 3).

4-Hydroxy-2,4’-dimethoxydihydrochalcone (2): White amorphous powder; ^1^H and ^13^C data were recorded at 400 and 100 MHz in CDCl_3_, respectively. The ^1^H and ^13^C NMR spectral data of compound 2 were closely related to compound 1 (Table 4 and Fig. 3), except for the absence of methoxy group at 6-OCH_3_ and the appearance of 1,2,4-trisubstituted aromatic proton at *δ* 7.07 (1H, d, *J* = 8.2 Hz, H-6), *δ* 6.44 (1H, d, *J* = 2.3 Hz, H-3), and *δ* 6.41 (1H, dd, *J* = 8.2, 2.3 Hz, H-5). The location of H-6 was assigned by HMBC spectra (Table 4) in which the aromatic proton H-6 (*δ* 7.07) correlated with carbon C-2 (*δ* 159.5), C-3 (*δ* 98.7), C-4 (*δ* 158.4) and C-β (*δ* 18.6). These spectral data confirm compound 2 as 4-Hydroxy-2,4’-dimethoxydihydrochalcone. It provides identical spectral data with those described in a previous report [22].

**Fig. 4:**
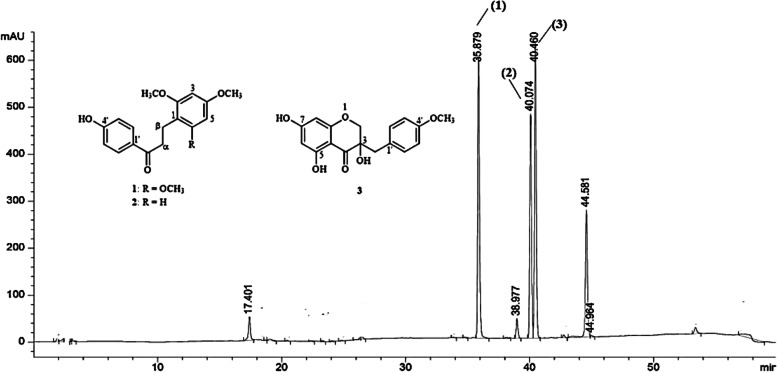
HPLC chromatogram of three isolated compounds of the ethanolic extract of Prasachandaeng remedy

Eucomol (3): Colorless crystal; ^1^H and ^13^C data were recorded at 500 and 125 MHz in CDCl_3_, respectively (Table 5 and Fig. 4). The NMR spectrum of compound 3 showed one methoxyl group at *δ* 3.80 (s, 4´-OCH_3_) and three hydroxyl group at *δ* 11.29 (s, 5-OH), and 3.40 (s, 3-OH). The para-disubstituted aromatic proton was present at *δ* 7.13 and 6.86 (each d, *J* = 8.3 Hz, H-2´, H-6´ and H-3´, H-5´, respectively), and *meta* proton at *δ* 6.04 (s, H-8) and 6.01 (s, H-6). The AB system of methylene proton at C-2 appeared at *δ* 4.19 and 4.07 (d, *J* = 11.1 Hz). In addition, the aliphatic methylene proton at C-9 was shown at *δ* 2.94 (2H, d, *J* = 7.0 Hz). In the HMBC spectra (Table 5), the methylene proton H-2 (*δ* 4.19 and 4.07) correlated with C-3 (*δ* 72.2), C-4 (*δ* 198.1), C-8a (*δ* 163.0) and C-9 (*δ* 40.6), the methylene proton H-9 (*δ* 2.94) correlated with C-3 (*δ* 72.2), C-4 (*δ* 198.1), C-1´ (*δ* 126.0) and C-2´ (*δ* 131.5), the aromatic proton H-6 (*δ* 6.01) correlated with C-4a (*δ* 100.5) and C-8 (*δ* 97.0), and the aromatic proton H-2´ (*δ* 7.13) correlated with C-3´ (*δ* 113.7), C-4´ (*δ* 158.8) and C-6´ (*δ* 131.5). These spectral data identified compound 3 as Eucomol ((3*S*)-3,5,7-trihydroxy-3-[(4-methoxyphenyl)methyl]-*2H*-chromen-4-one, which structure has been described in the literature [23]. Eucomol (3) showed potent cytotoxicity against KKU-M156 and HepG2 with the IC_50_ values of 7.12 ± 0.56 and 25.76 ± 1.56 µg/mL, respectively (Table 3). The structure of Eucomol differs from that of the classical isoflavones by the insertion of a carbon atom into the skeleton.

**Table 5:** NMR spectral data (500 MHz for ^1^H and 125 MHz for ^13^C) of compound 3

**Position**	***δ***_**C**_	***δ***_**H**_** (*J* in Hz)**	HMBC (*δ*_H_ → *δ*_C_)
2	71.7	4.19 (d, 11.1) 4.07 (d, 11.1)	C-3, C-4, C-8a, C-9
3	72.2		
4	198.1		
5	164.1		
6	95.7	6.01 (d, 6.01)	C-4a, C-8
7	165.3		
8	97.0	6.04 (s)	C-4a, C-6, C-7
9	40.6	2.94 (d, 7.0)	C-3, C-4, C-1′, C-2′
1′	126.0		
2′	131.5	7.13 (d, 8.3)	C-3′, C-4′, C-6′,
3′	113.7	6.86 (d, 8.3)	C-1′, C-4′, C-5′
4′	158.8		
5′	113.7	6.86 (d, 8.3)	C-1′, C-3′, C-4′
6′	131.5	7.13 (d, 8.3)	C-2′, C-4′, C-5′
4a	100.5		
8a	163.0		
4′-OCH_3_	55.2	3.80 (s)	C-4′
3-OH	-	3.40 (s)	C-2, C-4, C-4a,
5-OH	-	11.29 (s)	C-4, C-4a C-5

### Quantitative determination of the isolated compounds in PSD remedy

The contents of the three isolated compounds, Loureirin B (1), 4-Hydroxy-2,4’-dimethoxydihydrochalcone (2), and Eucomol (3), were simultaneously determined by a HPLC method. The three isolated compounds were the major chemical constituents of PSD95. The contents of each isolated compounds were calculated against its corresponded calibration curve which showed the r^2^ value greater than > 0.99. (1) showed the highest content (28.71 ± 1.22 mg/g) followed by (3) (24.81 ± 0.17 mg/g) and (2) (18.67 ± 0.14 mg/g), respectively. The chemical constituent contents and HPLC chromatogram of marker compounds are shown in Fig. 3 and 4.

### In vitro cytotoxicity of PSD remedy and plant ingredients

All crude extracts and isolated compounds exhibited significant differences from the anti-cancer drug vincristine sulfate (^****^p < 0.0001) in their cytotoxic activity against HepG2, KKU-M156, and HaCaT. The EtOH extract of PSD remedy (PSD95) exhibited potent cytotoxicity against hepatocellular carcinoma (HepG2) and cholangiocarcinoma (KKU-M156) with IC_50_ values of 10.45 ± 1.97 (SI = 5.30) and 4.53 ± 0.74 (SI = 12.25) µg/mL, respectively. In addition, PSD95 exhibited moderate cytotoxicity against HaCaT with IC_50_ values of 55.45 ± 1.73 µg/mL. Some plant ingredients also exhibited strong cytotoxicity against HepG2, i.e., 95% EtOH extract of *C. sappan* (CS95), *D. cochinchinensis* (DC95), *K. galanga* (KG95), *L. chuanxiong* (LC95), *M. siamensis* (MS95), *M. ferrea* (MF95), and *M. fragrans* (MYF95) with IC_50_ values of 6.44 ± 0.54 (SI = 6.7), 7.72 ± 1.87 (SI = 5.2), 7.81 ± 2.39 (SI = ND), 11.87 ± 4.43 (SI = ND), 5.67 ± 0.32 (SI = 9.0), 7.10 ± 0.16 (SI = 7.3), and 5.67 ± 0.32 (SI = 4.6) µg/mL, respectively. The chemotherapeutic drug (vincristine sulfate) exhibited cytotoxicity against HepG2, KKU-M156, and HaCaT with IC_50_ values of 0.012 ± 0.0005 (SI = 0.00058), 0.0026 ± 0.001 (SI = 0.0026), and 0.000007 ± 0.00 µg/mL, respectively. The summarized results of cytotoxic activity are shown in Table 6. The SI index of greater than > 3 shows a good selectivity index of cytotoxic activity using the SRB assay [15, 16]. The selectivity index (SI) in our study is shown in Table 6. The results indicate that all crude extracts did not show cytotoxicity towards the human non-cancerous cell line.

**Table 6:** IC_50_ µg/mL ± SEM of cytotoxicity of PSD remedy and its ingredients against two types of cancer cell lines and one non-cancerous cell line using Sulforhodamine B (SRB) assay (*n* = 3)

Plant species	Code	%Yield (w/w)	IC_50_ µg/mL ± SEM and Selective index (SI)		
			**HepG2**	**KKU-M156**	**HaCaT**
*Bouea macrophylla* Griff	BM95	3.52	36.15 ± 0.54^****^ (SI = ND)	60.53 ± 1.08^****^ (SI = ND)	> 100^****^
*Caesalpinia sappan* L	CS95	8.17	6.44 ± 0.54^****^ (SI = 6.7)	4.77 ± 0.57^****^ (SI = 9.0)	43.14 ± 1.46^****^
*Citrus aurantiifolia* (Christm.)	CA95	2.63	92.96 ± 2.22^****^ (SI = ND)	> 100^****^ (SI = ND)	> 100^****^
*Dracaena cochinchinensis* (Lour.) S.C. Chen	DC95	10.45	7.72 ± 1.87^****^ (SI = 5.2)	5.27 ± 5.01^****^ (SI = 7.7)	40.47 ± 0.39^****^
*Heliciopsis terminalis* (Kurz)	HT95	1.99	44.50 ± 1.01^****^ (SI = ND)	43.42 ± 0.29^****^ (SI = ND)	> 100^****^
*Jasminum sambac* (L.) Aiton	JS95	8.59	95.01 ± 0.41^****^ (SI = ND)	85.77 ± 2.25^****^ (SI = ND)	> 100^****^
*Kaempferia galanga* L	KG95	3.49	7.81 ± 2.39^****^ (SI = ND)	32.95 ± 3.75^****^ (SI = ND)	> 100^****^
*Ligusticum chuanxiong* Hort	LC95	8.50	11.87 ± 4.43^****^ (SI = ND)	43.55 ± 4.02^****^ (SI = ND)	> 100^****^
*Mammea siamensis* T. Anderson	MS95	19.41	5.67 ± 0.32^****^ (SI = 9.0)	7.52 ± 1.05^****^ (SI = 6.8)	51.20 ± 0.68^****^
*Mesua ferrea* L	MF95	11.57	7.10 ± 0.16^****^ (SI = 7.3)	27.17 ± 2.86^****^ (SI = 1.9)	51.99 ± 0.37^****^
*Myristica fragrans* Houtt	MYF95	2.88	5.67 ± 0.32^****^ (SI = 4.6)	5.02 ± 3.14^****^ (SI = 5.2)	26.32 ± 2.67^****^
*Nelumbo nucifera* Gaertn	NN95	8.08	> 100^****^ (SI = ND)	> 100^****^ (SI = ND)	> 100^****^
Prasachandaeng remedy	PSD95	13.62	10.45 ± 1.98^****^ (SI = 5.3)	4.53 ± 0.74^****^ (SI = 12.2)	55.45 ± 1.73^****^
Vincristine sulfate	-	-	0.012 ± 0.0005 (SI = 0.00058)	0.0026 ± 0.001 (SI = 0.0026)	0.000007 ± 0.00

*ND* Not detected, *SI* Selective index calculated by IC_50_ of normal cells /IC_50_ of cancer cells. Data were presented as mean ± SEM and analyzed by one-way ANOVA and Dunnett’s multiple comparison tests. Significant different presented the ^****^ *p* < 0.0001 compared with a standard drug (Vincristine sulfate) in corresponding cell line

### Determination of LPS-induced TNF-α production in RAW 264.7 cells

The DC95 and PSD95 were investigated for anti-inflammatory activity on TNF-α production in the murine macrophage cell line (RAW 264.7). The results are shown in Table 7. The DC95, PSD95 and acetaminophen (ACP) exhibited anti-inflammatory activity by inhibition of TNF-α production with the percentage values of 71.133 ± 2.806, 45.083 ± 1.814, and 18.657 ± 1.925%, respectively. The DC95 and PSD95 showed significant difference from acetaminophen (*p* > 0.05) in their anti-TNF-α production. The results also showed that DC95 and PSD95 exhibited inhibitory activity on TNF- α production 4, and 2.5-fold, respectively, higher than the standard anti-pyretic / analgesic drug, acetaminophen.

**Table 7:** Anti-inflammatory on LPS-induced tumor necrosis factor-alpha production of *D.cochinchinensis* and PSD remedy

Plant species	CODE	Inhibition of TNF-α production	
		**%Inhibition and [%cytotoxicity] at conc. 100 µg/mL**	**IC**_**50**_** µg/mL**
*D.cochinchinensis*	DC95	71.133 ± 2.806^****^ [25.676 ± 3.451^*^]	82.070 ± 7.570^*^
Prasachandaeng remedy	PSD95	45.083 ± 1.814^***^ [18.986 ± 0.460^*^]	> 100
Acetaminophen	ACP	18.657 ± 1.925 [59.222 ± 7.693]	> 100

(-) Indicates not tested. Data were analyzed by one-way ANOVA and Dunnett’s multiple comparison tests. Significant difference (^*^) is when ^*^p < 0.05, ^***^ *p* < 0.001, and ^****^ *p* < 0.0001 in comparison with an acetaminophen (ACP) (*n* = 3)

## Discussion

In this study, the ethnopharmacological wisdom of TTM for treating toxic (chronic) fever starting with the need to reduce high temperature (Pit—Ta; fire element) provided initial guidance). According to the TTM, PSD remedy has bitter and cold flavors that can reduce toxic fever. Furthermore, the ingredients exhibited several flavors, i.e., astringent, fragrant, spicy. These combinations of ingredients and the amelioration of related symptoms of chronic diseases are linked. The flavors of PSD ingredients and their bioactivity are shown in Table 8. The 95% ethanolic extract of PSD remedy extract (PSD95) exhibited strong cytotoxic activity against two types of human cancer cell lines, i.e., hepatocellular carcinoma cell line (HepG2) and cholangiocarcinoma cell line (KKU-M156). Interestingly, its ingredients of PSD remedy, i.e., *C.sappan* (CS95), *D.cochinchinensis* (DC95), *M.siamensis* (MF95), and *M. fragrans* (MYF95) also exhibited strong cytotoxic activity against cholangiocarcinoma cell line (KKU-M156) with IC_50_ values less than 10 µg/mL, respectively. The previous study demonstrated that the 70% ethanolic extract of *C.sappan* showed cytotoxic activity against hepatocellular carcinoma (HepG2) cell line [24]. Additionally, the *Streptomyces* sp. HUST012 (SPE-B5.4) was isolated from the heartwood of *D.cochinchinensis* resulted in potent cytotoxic activity against hepatocellular carcinoma cell line (HepG2) with an IC_50_ value of 0.23 µg/mL [25]. The results of this study were in accordance with previous study that demonstrated the *C.sappan* and *D.cochinchinensis* exhibited cytotoxic activity against hepatocellular carcinoma (HepG2) with the IC_50_ values less than 20 µg/mL. On the other hand, the extract of *M.ferrea* showed comparably modest cytotoxic activity using MTT assay against cholangiocarcinoma cell line (CL-60) with IC_50_ value of 48.23 µg/mL [26].

**Table 8:** The flavors of herbal ingredients and relationship between the usage of Thai traditional medicine (TTM) and evidence-based approach of herbal medicine of PSD remedy and its ingredients

Plant species (Family)	Thai name	Flavor	TTM used^a,b^	Evidence-based approach of herbal medicine
*Bouea macrophylla* Griff. (Anacardiaceae)	Ma-Prang	Flavorless	Antipyretic^a^, diuretic^b^	Antioxidant [27]
*Caesalpinia sappan* L. (Leguminosae)	Faang	Astringent	Anti-inflammatory^a^, wound healing^a^, detoxification^a^, increase blood circulation^a^, blood tonic^a^, cardiotonic^a^, chicken pox^b^	Anti-inflammatory, Antioxidant, Cytotoxic [24, 28, 29]
*Citrus aurantiifolia* (Christm.) Swingle (Rutaceae)	Ma-Nao	Flavorless	Antipyretic^a^, detoxification^a^, increase blood circulation^a^, common cold influenza^a^, diuretic^b^	Cytotoxic, Anti-inflammatory [30–32]
*Dracaena cochinchinensis* (Lour.) S.C. Chen (Dracaenaceae)	Chan-Daeng	Bitter&cold	Antipyretic^a^, anti-inflammatory^a^, wound healing^a^, detoxification^a^, increase blood circulation^a^, blood tonic^a^	Anti-inflammatory, Cytotoxic, Antipyretic [25, 33, 34]
*Heliciopsis terminalis* (Kurz) Sleumer (Proteaceae)	Mhuad-Kon	Flavorless	Common cold influenza^a^, diuretic^b^	Anti-inflammatory [10]
*Jasminum sambac* (L.) Aiton (Oleaceae)	Ma-Li	Fragrant	Antipyretic^a^, cardiotonic^a^, sleep disorder^b^, headache^b^	Anti-inflammatory, and antipyretic [35]
*Kaempferia galanga* L. (Zingiberaceae)	Por-Hom	Spicy	Common cold influenza^a^, reducing the dyspepsia^a^, increase blood circulation^b^	Anti-inflammatory [36]
*Ligusticum chuanxiong* Hort. (Umbelliferae)	Khod-Hua-Bua	Spicy	Anti-inflammatory^a^, wound healing^a^, increase blood circulation^b^	Cytotoxic [37]
*Mammea siamensis* T. Anderson. (Calophyllaceae)	Sa-Ra-Pee	Fragrant	Antipyretic^a^, cardiotonic^a^	Cytotoxic, Anti-inflammatory [38, 39]
*Mesua ferrea* L. (Calophyllaceae)	Boon-Nak	Fragrant	Blood tonic^a^, antipyretic^a^ headache^a^, cardiotonic^a^	Cytotoxic [26, 40]
*Myristica fragrans* Houtt. (Myristicaceae)	Chan-Thet	Fragrant	Enhancing immune function^a^, blood tonic^a^, cardiotonic^a^, antipyretic^a^, headache^a^, dyspepsia^a^	Cytotoxic, Anti-inflammatory [41–43]
*Nelumbo nucifera* Gaertn. (Nelumbonaceae)	Bua-Luang	Fragrant	Cardiotonic^a^, antipyretic^a^	Cytotoxic, Antipyretic [44, 45]
Prasachandaeng remedy	-	Bitter&cold	Reduction of fever (chronic fever), and treatment of aphthous stomatitis	Anti-inflammatory, antipyretic [10, 46]

(-) Indicates the data not shown. ^a^Thai traditional medical textbook, The Rehabilitation Foundation for Thai Traditional Medicine and Ayuraved Thamrong School, 2007. ^b^The usages according to Thai traditional doctor

Compound 1 exhibited cytotoxicity against HepG2 and KKU-M146 with the IC_50_ values of 20.02 ± 0.46 and 21.26 ± 3.17 µg/mL, respectively (Table 3). Current evidence indicates that retrodihydrochalcones can exert antiproliferation activity against human cancer cell lines when they carry hydroxy substituents in appropriate positions. The active compounds share two para-hydroxybenzene rings connected by a chain of three carbon atoms. This is in sharp contrast to isoflavones which are regarded as analogs of dihydroxystilbene in which two para-hydroxybenzene rings are connected via a chain of two carbon atoms. These findings regarding the structure–activity relationship and antiproliferation activity require further investigation [21]. Our current findings demonstrate cytotoxicity of 4-Hydroxy-2,4’-dimethoxydihydrochalcone (2) against HepG2 and KKU-M146 with the IC_50_ values of 20.71 ± 0.49 and 33.21 ± 2.10 µg/mL, respectively. There has been no previous report on cytotoxic activity of 4-Hydroxy-2,4’-dimethoxydihydrochalcone (2) against cancer cell lines. This is also the first scientific evidence of its cytotoxic activity against cancer cell lines in comparison with a non-cancerous cell line.

Eucomol (3) has three OH groups that can increase the bioactivity [23]. This is the first report of isolation of Eucomol (3) from the heartwood of an ethanolic extract of *D. cochinchinensis*. In our investigations, we have discovered flavonoids that are an important class of natural products. They belong to a class of plant secondary metabolites having a polyphenolic structure widely found in fruits, vegetables, and herbs. There are several well characterized bioactivities of flavonoids such as antioxidant, anti-inflammatory, and anti-carcinogenic properties [47]. The results of this study are in accordance with a previous study which demonstrated that *D. cochinchinensis* exhibited cytotoxic activity against hepatocellular carcinoma (HepG2) with IC_50_ values less than < 20 µg/mL [25]. This is the first report of PSD95 and DC95 on anti-TNF-α production in RAW264.7. Both crude extracts showed the % inhibition of anti-inflammatory activity via TNF-α production higher than the positive control acetaminophen. Therefore, these results support the use of PSD95 and DC95 for treating chronic fever based on their ability to inhibit the pro-inflammatory cytokine-related carcinogen than a well established drug used clinically. However, we investigated the pharmacology of PSD remedy in comparison with a drug known to possess antipyretic activity in animal models [46]. Quality control of the chemical contents of PSD95 with a validated HPLC method was determined. The study provided preliminary data on the major chemical constituents of the PSD remedy. However, further studies on molecular docking of the pure compounds and additional biological and pharmacological characterization are warranted.

## Conclusion

The scientific evidence detailed in these investigations suggests that the three isolated compounds discovered had anti-cancer proliferative activity. The PSD remedy exhibited potent cytotoxic activity against hepatocellular carcinoma (HepG2) and cholangiocarcinoma (KKU-M156). In fact, PSD remedy exhibited a greater anti-inflammatory activity as measured by inhibition of TNF-α production than acetaminophen. The results of this study support the Thai traditional wisdom that the uses the herbal combination as ingredients in traditional remedies may be effective as medicines for cancer, at least in part through their anti-inflammatory, and antipyretic activities. As a result of this study, the Thai traditional practitioners and folk doctors that use PSD remedy for toxic fever in liver and bile duct cancer patients also have additional scientific evidence underlying the rationale of their continued clinical use.

## Data Availability

Dataset of this manuscript has not been deposited in any reposition. All datasets and materials are available from the corresponding author upon reasonable request.

## Abbreviations

ATCC: American type culture collection
CHCl_3_: Chloroform
CC: Column chromatography
DMEM: Dulbecco’s modified eagle medium
DMSO: Dimethylsulfoxide;
ELISA kit: Enzyme-linked immunosorbent assay
EtOAC: Ethyl acetate
EtOH: Ethanol
FBS: Fetal bovine serum
HaCaT: Human keratinocyte cell line
HCl: Hydrochloric acid
HepG2: Hepatocarcinoma cell line
HPLC: High performance liquid chromatography
KKU-M156: Cholangiocarcinoma cell line
LPS: Lipopolysaccharide
MEM: Minimum essential media
MeOH: Methanol
NMR: Nuclear magnetic resonance
PBS: Phosphate buffered saline
PSD: Prasachandaeng
SEM: Standard error of means
SI: Selectivity index
SRB assay: Sulforhodamine B assay
TCA: Trichloroacetic acid
TNF-α: Tumor necrosis factor-alpha
TLC: Thin layer chromatography
TTM: Thai traditional medicine
VLC: Vacuum liquid chromatography
